# Recovery of Biomolecules from Food Wastes — A Review

**DOI:** 10.3390/molecules190914821

**Published:** 2014-09-17

**Authors:** Antonietta Baiano

**Affiliations:** Department of Agricultural Sciences, Food and Environment, University of Foggia, Via Napoli, 25–71122 Foggia, Italy; E-Mail: antonietta.baiano@unifg.it; Tel.: +39-0881589349

**Keywords:** biomass, by-products, dietary fiber, food waste, nutraceutical products, phenolic compounds, recovery

## Abstract

Food wastes are produced by a variety of sources, ranging from agricultural operations to household consumption. About 38% occurs during food processing. At present, the European Union legislation encourages the exploitation of co-products. This valorisation can be achieved through the extraction of high-value components such as proteins, polysaccharides, fibres, flavour compounds, and phytochemicals, which can be re-used as nutritionally and pharmacologically functional ingredients. Extraction can proceed according to solid-liquid extraction, Soxhlet extraction, pressurized fluid extraction, supercritical fluid extraction, ultrasound-assisted extraction, microwave-assisted extraction, pulsed electric field extraction, and enzyme-assisted extraction. Nevertheless, these techniques cannot be used indiscriminately and their choice depends on the type of biomolecules and matrix, the scale processing (laboratory or industrial), the ratio between production costs and economic values of the compounds to be extracted. The vegetable wastes include trimmings, peelings, stems, seeds, shells, bran, residues remaining after extraction of oil, starch, sugar, and juice. The animal-derived wastes include wastes from bred animals, wastes from seafood, wastes from dairy processing. The recovered biomolecules and by-products can be used to produce functional foods or as adjuvants in food processing or in medicinal and pharmaceutical preparations. This work is an overview of the type and amounts of food wastes; food waste legislation; conventional and novel techniques suitable for extracting biomolecules; food, medicinal and pharmaceutical uses of the recovered biomolecules and by-products, and future trends in these areas.

## 1. Food Wastes

Food wastes are produced throughout all the food life cycle. Excluding the agricultural food losses, up to 42% of them is produced by households, 38% occurs during food processing, and 20% is distributed along the whole chain [1]. Concerning the food industry, wastes derive from the processing of raw vegetable and animal materials into foodstuffs, which generally consists in the extraction or separation of the nutritionally portion from the remains having little nutritional value or inedible components [2]. The food industry produces large amounts of wastes. In Europe, the wastes generated from the production of foods were about 222 million tons in 2004 [3]. Food wastes derive, in a decreasing order, from the following sectors: vegetables and fruits; milk; meat; fish, and wine. In the Italian food industry, waste amounted to an average of 2.6% of the total, equal to almost 1.9 million tons of food (excluding the beverage sector). In order to give an idea of the amount of the generation of the different waste streams, Table 1 shows the quantity of waste produced and the % ratio between amount of waste and amount of production of each industrial sector [4]. Most food waste derives from the drink industry (26%), followed by the dairy and ice cream industry (21.3%), the production and preservation of fruits and vegetables (14.8%), the manufacture of grain and starch products (12.9%), the production, processing and preservation of meat products (8%), the manufacture of vegetable and animal oils and fats (3.9%), the production and preservation of fish and fish products (0.4%). The wastes deriving from the manufacture of other food products amount to 12.7%. The discarded products are usually managed as refuse or used to produce animal feed. Until a few decades ago, food wastes were considered neither a cost nor a benefit (they were used as animal feed or brought to landfills or sent for composting). This attitude has recently changed for several reasons including the growing environmental concerns in the European Union; the demand for controls to minimize the impact of waste on human health, which is bringing about more stringent regulations; the high disposal costs that are eroding the already low profits of the food industry and the growing awareness of the benefits deriving from potentially marketable components present in foods wastes and co-products [5].

**Table 1:** Estimate of waste in the food industry [4].

Industrial Sector	Amount of Waste (000 t)	Waste (%)
Production, processing, and preserving of meat and meat products	150	2.5
Production and preserving of fish and fish products	8	3.5
Production and preserving of fruits and vegetables	279	4.5
Manufacture of vegetable and animal oils and fats	73	1.5
Dairy products and ice cream industry	404	3
Production of grain and starch products	245	1.5
Manufacture of other food products	239	2
Drinks industry	492	2
Total	1890	2.6

Concerning legislation, the EU has established that landfilling is no longer sustainable. The key legal instrument is represented by the Landfill Directive [6], which was aimed at reducing biodegradable waste (food waste included) disposed to landfill (35% of the 1995 level by 2016). The Hazardous Waste Directive [7], which also referred to cereal and vegetable food-processing wastes, contains criteria for the elaboration of a hazardous waste list/waste catalogue, and requires accurate hazardous waste management planning. The Waste Framework Directive [8] highlights the relative priority of the different methods of managing waste, a concept that will affect the future legislation and policies. In particular, the waste management hierarchy includes, in descending order of priority, the following actions: waste prevention; re-use; recycling; other recovery (e.g., energy recovery); and, lastly, disposal. According to this list, co-product exploitation must be encouraged in order to prevent that food waste should be regarded as waste. In addition, among the policies created in Europe to promote the prevention of food waste, the bio-economy strategy must be cited [9]. According to the European Commission [8], the Europe Countries must change their approach to production, consumption, processing, storage, recycling, and disposal of biological resources. In this light, developments in bio-economy research and innovation will improve the management of the biological resources. They will also open new markets in food and bio-based products. The European Commission also highlighted the need for continuous and increasing support from public funding and private investment and the contribution of the bio-economy to improve coherence between national, European, and international research and development efforts.

Regarding the potentially marketable components present in foods wastes and co-products, the aim is to exploit high value components such as proteins, polysaccharides, fibers, flavor compounds, and phytochemicals as nutritionally and pharmacologically-functional ingredients. For the valorization of co-products, the legal permissibility of their use is fundamental. In order to assess the permissibility of a product used as an input substance (*i.e.*, a substance used in the food processing), this input substance has to be qualified and the intended use of the output substance must be known since they determined the legislation applied [10]. If the output is a food/food ingredient, the input must be a food/food ingredient in agreement with the Article 2 of the EC Regulation N. 178/2002 [11]. According to this article, food is “any substance or product, whether processed, partially processed or unprocessed, intended to be, or reasonably expected to be ingested by humans” and “includes drink, chewing gum and any substance, including water, intentionally incorporated into the food during its manufacture, preparation or treatment”. Instead, the definition of “food” does not include substances such as feed, live animals unless they are prepared for placing on the market for human consumption, plants prior to harvesting, medicinal products, cosmetics, tobacco and tobacco products, narcotic or psychotropic substances, residues and contaminants. The qualification of a certain substance as food has great implications, since it means that generic and specific food legislation must be applied. Input and output substances can be also defined as “novel” according to the EC Regulation N. 258/97 [12]. The novel foods are foods and food ingredients that were not used before 15 May 1997 within the European Community for human consumption to a significant degree. In order to ensure the protection of human health, novel foods must undergo a safety assessment before their placement on the European Union market. In particular, who is aimed to place a substance as a novel food has to submit a request to the Member State in which the product will be sold for the first time and to wait for the authorization. The request must be submitted according to the EC Recommendation N. 97/618 [13].

The potentially marketable components present in foods wastes and co-products need to be separated from the matrix through combined (biochemical, chemical and physical) approaches for selective extraction and modification of the targeted components and changed into higher value food products or additives. These operations must be performed avoiding microbiological hazards and ensuring that the final products comply with the existing food regulations and meet consumer liking. Acceptability is a very important characteristic since the potential customers could perceive the use of co-product valuable components as a reduction of the food quality. The optimization of the extraction conditions using conventional and emerging techniques and the use of the recovered biomolecules and by-products have been the object of several research activities. Examples of them include the works of Baiano *et al.* [14,15], aimed to investigate: (a) the single and interactive effects of process variables on yield and antioxidant concentrations of aqueous extracts obtained through conventional and microwave-assisted extraction from vegetable solid wastes of cauliflowers, celery, chicory and asparagus; (b) the possibility of replacing the ingredient “flour” with spent biomass derived from the production of beers in order to produce functional breads. Other studies are currently in progress.

The extraction of the high-value components must be economically feasible to perform. This objective can be achieved by separating the components of interests through individual and/or combined physical and biochemical approaches so to provide a range of components, from high value to low value, all of which would contribute to achieving whole-waste exploitation. In the case of co-products unsuitable for food exploitation, they must be used as energy sources via fermentation, biogas production, and composting [16]. These statements clearly highlight that the valorization of food wastes can be achieved through an integrated bio-refinery approach, in order to produce bioactive molecules for pharmaceutical, cosmetic, food, and non-food applications. In addition, the exploitation of food co-products and wastes through the production of high valuable foods could require an evaluation under regulation governing novel foods and novel food ingredients [12].

In Europe, large-scale facilities valorizing different food waste streams for biomolecules recovery have been established. For example, the Bio-based Industries Joint Undertaking (BBI JU) is a public-private partnership between the European Commission and the Bio-based Industries Consortium (BIC), whose aim is to bring together all relevant stakeholders (ranging from primary production to end-users) to establish innovative bio-based industries. The Joint Undertaking work plan has a total budget of €3.705 billion, of which almost 75% will be contributed by the Bio-based Industries Consortium. In 2014, the Bio-based Industries JU’s first Call for Proposals includes topics for several value chains. With reference to the facilities aimed to the recovery of biomolecules from food wastes, the potentially fundable research and innovations actions concern: The isolation of sugars from rejects and side streams of pulp and paper industry and their conversion into added value bio-products such as carbohydrate derivatives (e.g., alcohols, polyols, small organic acids); the development of industrially viable processing concepts for the valorization of protein products from plant residues (e.g., from bioethanol or cereal starch processing industries) fulfilling market requirements in the food segment; the use of meso-organisms to convert agro-based residues (e.g., cereal residues, sugar residues or oil rich plant residues) into highly bioactive protein-rich, lipid-rich and chitin-rich bioactive compounds; the production of functional additives from residues from the agro-food industry [17].

## 2. Extraction Techniques

The extraction conditions are extremely important, due to their effects on the release of compounds from the matrix into the medium [18]. Hence an overview of the main extraction systems is reported in this section.

### 2.1. Solid-Liquid Extraction

Solid-liquid extraction allows soluble components to be removed from solids using aqueous organic solvents, alone or mixed together. The choice of the solvents must be carefully made in order to eliminate or minimize matrix interferences [19], while the experimental parameters (temperature, time, pH, solid-to-liquid ratio, particle size, stirring, solvent polarity) must all be optimized in order to obtain a quantitative extraction of the molecules of interests. The disadvantages of this type of extraction are the requirement for expensive, partly toxic, inflammable, explosive, and hazardous organic solvents and the long times needed [20].

Solid-liquid extraction has a great number of industrial applications. For example, in the herbal and other food preparation industries, it is currently applied when a vegetable matrix requests extraction for further processing. The actual and future research direction is represented by the use of cheap and non-toxic solvents (*i.e.*, water) to be combined with other mild extraction techniques.

### 2.2. Soxhlet Extraction

Compared to solid-liquid extraction, the advantages include the repeated washing of the matrix with fresh solvent, the higher possible solubilization of the analyte due to the use of hot solvent, and the possibility to apply it to many food matrices. These characteristics make the systems cheaper than other techniques, allowing strong economy in terms of time, energy, and consequently financial inputs. At a small scale, Soxhlet extraction is employed as a batch process, but it can be converted into a continuous procedure that, on medium or large scale, allows further economies.

The main disadvantages are the same as those of the traditional solid-liquid extraction and the risk of degradation for temperature sensitive compounds [21]. It is generally used as an analytical technique in food testing and has only a limited application in food processing (extraction of essential oils and volatile compounds from botanical species).

Soxhlet extraction has better performance than other conventional extraction techniques except for the extraction of thermolabile compounds. It also has more advantages than novel techniques such as ultrasound-assisted, microwave-assisted, supercritical fluid, and accelerated solvent extractions in terms of industrial applications, reproducibility, efficiency, and extract manipulation. The conventional Soxhlet extraction is become the starting point for the development of a variety of modifications (high-pressure, automated, ultrasound-assisted, and microwave-assisted Soxhlet extraction) intended to shorten the leaching times with the use of auxiliary forms of energy and automating the extraction operation.

### 2.3. Pressurized Fluid Extraction and Supercritical Fluid Extraction

Pressurized fluid extraction is similar to Soxhlet extraction, except that the solvents used near their supercritical region. Under such conditions, the high temperature enables higher solubility and higher rate of solute diffusion in the solvent, while the application of high pressure maintains the solvent below its boiling point, thereby allowing a high penetration of the solvent into the sample. These conditions reduce the solvent volumes (15–40 mL) needed and shorten the extraction times (15–20 min).

A fluid is called supercritical when its temperature and pressure are higher than the corresponding critical values of the chosen fluid. The critical values mark the end of the vapor-liquid coexistence curve. The use of supercritical fluid extraction consists in the separation of the extractant from the matrix (which is usually solid, but can also be a liquid) using supercritical fluids as the extracting solvent. Carbon dioxide is the most used supercritical fluid, sometimes modified by co-solvents such as ethanol that change its polarity. The choice of supercritical CO_2_ is due to its moderate critical conditions (31.1 °C and 73.8 MPa), absence of toxicity, and chemical stability. Some of the advantages of supercritical fluid extraction are: solvating powers similar to liquid organic solvents, high solute diffusivities, lower viscosity, and lower surface tension; and the possibility of adjusting the solvating power by changing pressure or temperature. Also, the separation of the solutes from the fluid is easily facilitated. Moreover, CO_2_ is cheap, safe, and easy to recycle and the use of supercritical CO_2_ gives cleaner extracts than other conventional extraction techniques.

Supercritical fluid extraction has extensive application in the extraction of pesticides, environmental samples, foods and fragrances, essential oils, polymers and natural products. The biggest hurdle in the commercial application of this technique is its prohibitive capital investment. Commercialization of supercritical extraction processes is limited since this technique has been developed in isolation of other processing steps that are generally necessary to obtain a product. This limit can be overcome through integrated processing operations including: upstream fractionation (pre-processing of materials by solvent extraction or physical separations to produce a partial concentration); production of tailored feedstocks by microorganisms (for production of a wide range of biochemicals); pre-processing by chemical or enzymatic reactions (in order to break the bonds between the compounds of interest and other cellular structures); *in situ* processes (adsorption or reaction operations that are carried out under supercritical conditions). These integrated processing operations are being investigated or yet to be fully developed, but they represents the current emerging trends.

### 2.4. Ultrasound-Assisted Extraction

Ultrasound-assisted extraction has not only been used to extract bioactive compounds such as antioxidants and tocols, but also essential oils, steroids, and lipids from plants. Sound waves (frequencies higher than 20 kHz) travel in matter and involve expansion and compression cycles. Expansion pulls molecules apart while compression pushes them together. In a liquid, the expansion can create bubbles that grow and then collapse. Close to a solid boundary, cavity collapse is asymmetric and involves high-speed jets of liquid, which have strong impact on the solid surface [22]. Ultrasound induces a greater penetration of solvent into cellular materials, thus improving mass transfer, and also disrupts biological cell walls, thus facilitating the release of contents. Ultrasound frequency can have great effects on extraction yield and kinetics, depending on the nature of the plant material to be extracted [23]. In any case, the use of ultrasound allows a decrease in the temperature and pressure with respect to the extraction without ultrasound [24] and the apparatus is cheaper than that of microwave-assisted extraction. The main disadvantages of this type of extraction are the ultrasound attenuation exerted by the presence of a dispersed phase and due to differences in compressibility, heat capacity, and thermal diffusion between the droplets of the dispersed phase and the continuous primary phase; the reduced efficiency of the ultrasound in areas of the extractor located far from the emitter [25].

Ultrasound-assisted extraction is not an off-the-shelf technology. This means that it must be developed and scaled up for each application. Nevertheless, ultrasonic processing has the capability for large commercial scale-up and good payback on capital investment (generally less than 1 year) due to the availability of high amplitude/power units for large commercial operations; improved energy efficiency of the equipment, ease of installation and/or of retrofitting, competitive energy costs and low maintenance costs [26].

### 2.5. Microwave-Assisted Extraction

Microwaves are electromagnetic waves consisting of an electric and a magnetic field which oscillate perpendicularly to each other at frequencies ranging from 0.3 to 300 GHz. Microwave energy acts directly on molecules by ionic conduction and dipole rotation and thus only polar materials can be heated based on their dielectric constant [27]. For this reason, microwave-assisted extraction depends on the dielectric susceptibility of both solvents and matrices and is particularly promising for the extraction of compounds of medium to high polarity from a solid matrix. Since water within the plant matrix absorbs microwave energy, cell disruption is determined by an internal superheating, which also allows the separation of compounds of interest from the matrix, thus improving their recovery. Microwave extraction apparatus are divided into closed and open system. In a closed system, the extractions are carried out in a sealed-vessel under uniform microwave heating. In such a system, the high pressures and temperatures allow fast and efficient extraction. Open systems counter the shortcomings of closed system such as the safety issues and are more suitable for extracting thermo-labile compounds since they operate under milder conditions [28].

Microwaves are a powerful alternative to the traditional solid–liquid system when nutraceuticals must be extracted from plants for the following reasons: reduction of both extraction time and solvent usage and improved extraction yields. Microwave-assisted extraction is also comparable to supercritical fluid extraction due to its process simplicity and low cost, but is more expensive than ultrasound-assisted extraction.

Several modified microwave extraction systems have been developed. They include vacuum microwave-assisted extraction for the extraction of thermal sensitive compounds using mild operating conditions (in this case, the mass transfer mechanism of active compounds from the matter to the solvent occurs via the suction pressure), nitrogen-protected microwave-assisted extraction in which nitrogen is used to pressurize the extraction vessel and to avoid the oxidation of oxygen sensitive molecules during the extraction, ultrasonic microwave-assisted extraction in which the combined microwave and ultrasonic waves intensify the mass transfer mechanism as a consequence of the high energy which is suitable for breaking plant cells and eluting the active compounds into the extraction solvent, and dynamic microwave-assisted extraction where the extraction process is performed in a continuous and automatic manner and coupled to an on-line analytical step [28].

Microwave-assisted extraction is a promising technique in analytical laboratories but its use for the extraction of natural compounds on industrial scale is still limited. Paré [29,30] has patented the microwave-assisted extraction of essential oils.

### 2.6. Pulsed Electric Field Extraction

During the extraction of intracellular compounds, the cell wall and the cell membrane are natural barriers, which preserve the cell content and avoid their release. The application of a pulsed electric field to cells above a trans-membrane potential of about 1 V results in the formation of pores in the membrane (electroporation) [31]. This pore formation is due to polarisation of ions across the membrane, which compresses the electrically non-conductive membrane to the point of rupture [32], thus determining increases of the cell membrane permeability and hence the release of molecules in the medium already at the levels of 0.4–0.8 kV/cm [33]. The application of pulsed electric fields involves discharge of high voltage electric pulses for a few to several hundred microseconds into the cellular tissues placed or passed between two electrodes. Electroporation of the cellular tissue depends on parameters such as pulse duration (pulse width), number of pulses, and pauses between pulses. The pore formation can be reversible or irreversible, depending on the intensity of the treatment. When a low intensity treatment is applied, pores are small in comparison to the membrane area and the electric breakdown is reversible. Increasing the intensity and or the time of treatment results in the formation of large pores and generally irreversible permeabilisation.

From an energetic point of view, a pulsed electric field is an efficient process compared to thermal pasteurization, since it would add only 0.03–0.07 US$/L to the final food costs. Generation of high voltage pulses having sufficient peak power is the limitation in processing large quantities of fluid economically. The emergence of solid-state pulsed power systems removes this limitation, since it can be arbitrarily sized by combining switch modules in series and parallel [34]. Commercially, equipment with capacities of 10,000 L/h or more for preservation applications and in excess of 50,000 L/h for enhanced extraction or drying applications are available.

### 2.7. Enzyme-Assisted Extraction

The release of bioactive compounds from plant cells by cell disruption and extraction can be optimized using enzyme preparations either alone or in mixtures. Enzyme-assisted extraction is a promising alternative to conventional solvent-based extraction methods. It is based on the ability of enzymes to catalyze reactions with specificity and regioselectivity, under mild processing conditions, in aqueous solutions [35]. For these reasons, it is considered an environmentally friendly extraction method. Enzymes such as pectinases, cellulases, hemicellulases, *etc.* hydrolyze cell wall components thereby increasing cell wall permeability and the extraction yield of targeted compounds such as polysaccharides, oils, natural pigments, flavors, antioxidant, and medicinal compounds [36,37]. The enzymes used can derive from bacteria, fungi, animal organs, and vegetable extracts. In order to increase the extraction yields, operating conditions such as time, temperature, pH, and enzyme-to-substrate ratio must be optimized. Enzymes can be also used in a raw material pre-treatment step in order to reduce extraction times, minimize use of solvents and provide increased yield and quality. The technical limitations of this technique include the relatively cost for processing large volumes of raw material, the current availability of enzyme preparations that cannot completely hydrolyze plant cell walls, the difficulty to scale up enzyme-assisted extraction to industrial scale because enzymes behave differently as the environmental conditions (percentage of dissolved oxygen, temperature and nutrient availability) change.

## 3. Recovery of Biomolecules from Vegetable Wastes

These wastes include: vegetable and fruit trimmings; peelings, stems, seeds, shells; cereal residues (such as bran; residues remaining after deoiling); starch, sugar and juice extraction; off-spec, damaged, out-of-date, or returned products [9]. Biomolecules potentially extractable from the targeted wastes include sugars, polysaccharides, ethanol, proteins and derivatives, fibers, natural flavor compounds, phytochemicals.

Table 2 summarizes the information contained in this section in terms of extractable biomolecules, substrates, method of extraction, yield, and references.

The solid waste, often called “pomace”, is obtained by pressing of fruits or vegetables and can contain pulp, peels, seeds and, stones. For example, apple pomace is a by-product deriving from juice pressing. It is considered a source of dietary fibre (about 50% of dry weight) and phenolics (from 1200 to 4000 mg/kg dry weight), including flavanols (catechin, epicatechin, procyanidins), flavonols, hydroxycinnamates, and dihydrochalcones [38]. Kołodziejczyk *et al.* [39] were able to use apple pomace for the preparation of liquid and solid phenolic concentrates containing 2.5% and 33% of polyphenols, respectively, and for the preparation of dietary fiber (about 72% and 10% of total and soluble dietary fiber, respectively). The liquid concentrates were prepared starting from 1.5 kg of dried and ground pomace that was extracted in three batches with 7 L of 15%, 5 L of 70% and 3.5 L of 50% aqueous ethanol, respectively. The extracts from the second and the third batches were combined, the ethanol was evaporated, and the water extract was concentrated to 54.3° Bx. For the preparation of the solid concentrates, the extraction was performed as above, ethanol was evaporated, and the resulting water solution was adsorbed on ion exchange resin and then desorbed with 60% ethanol. Ethanol was evaporated, solution was concentrated and cooled down to 5 °C in order to remove crystallized quercetin, and solution was lyophilized. Dietary fiber was obtained from pomace extracted as above by drying in a hot air drier at 70 °C, and grinding to particle size below 0.5 mm.

The raw materials for pectin production are the dried press cake of apple juice manufacture (apple pomace, with 10%–15% extractable pectins) and the wet or dried peels and rags of citrus juice manufacture (citrus peel, with 20%–30% extractable pectins), and also sugar beet, sunflower heads and the wastes from the processing of tropical fruits. Pectin is extracted by treating the raw material with hot dilute mineral acid at pH 2. The hot viscous pectin extract is separated from the swollen and partly disintegrated residue by centrifugation and filtration. The clarified extract is brought to pH 4 and concentrated under vacuum prior to alcoholic precipitation, pressing and drying [16].

Essential oils are directly produced in juice production plants as a co-product from citrus. The main wastes include the citrus peels and residues from segments and seeds after pressing. They are rich in pectins and flavanones. Flavanones are generally extracted in alkaline water, then the extracts are acidified and the flavanones, which are slightly soluble in water, precipitate [16].

Tomato pomace includes dried and crushed skins (rich in lycopene and carotenes) and seeds of the fruit. Lycopene and β-carotene can be extracted by supercritical CO_2_ resulting in recoveries of up to 50% when ethanol was added [40].

Anthocyanins, usable as natural antioxidants or colorants, can be efficiently extracted from grape skins by heat treatment at 70 °C or by different novel technologies such as ultrasonics (35 KHz), high hydrostatic pressure (600 MPa, HHP), and pulsed electric fields (3 kV/cm). Under equal time of treatment (1 h), the total phenolic content was 50% higher in the extracts obtained through the novel technologies than in the control [41].

**Table 2:** Summary of the paragraphs 3 and 4: Molecule of interest, substrate from which it was extracted, extraction method and yield.

Extractable Biomolecule	Substrate	Extraction Method	Yield
Pectin	Apple pomace, Citrus peel, Sugar beet, Sunflower heads, wastes from tropical fruits	Solid-liquid extraction [16]	10%–15%, 20%–30%
Flavanones	Citrus peels and residues from segments and seeds after pressing	Solid-liquid extraction [16]	
Total and soluble dietary fibres	Apple pomace	Solid-liquid extraction [38]	72% and 10%
Phenolic compounds	Apple pomace	Solid-liquid extraction [39]	33%
Lycopene and β-carotene	Tomato pomace	Supercritical CO_2_ [40]	50%
Anthocyanins	Grape skins	Heat treatment at 70 °C, Ultrasonics, High hydrostatic pressure, Pulsed electric fields [41]	Variable
Caffeine	Green tea leaves	Supercritical fluid extraction [42]	97%
Essential oils (matricine, chamazulene and α–bisabolol	Chamomile	Supercritical fluid extraction [43]	28.08%, 0.05%, and 2.68%, respectively
Capsaicinoids and colour components	Chilli pepper	Supercritical fluid extraction [44]	66%–86% and 26%–34%, respectively
Oil	Rice bran	Supercritical fluid extraction [45]	24.65%
γ-oryzanol	Rice bran	Solid-liquid extraction [46]	1527–4164 mg/kg
β-glucans	Barley bran	Solid-liquid extraction [47]	
Lignans	Flaxseeds	Solid-liquid extraction [48]	
Phenolic acids	Wheat brans	Solid-liquid extraction, ultrasound assisted extraction, microwave-assisted extraction [23,49]	
Tocopherols, tocotrienols, sterols, and squalene	Palm fatty acid distillate	Liquid-liquid extraction [50,51,52]	
Phenolic antioxidants	Aqueous by-products from the palm oil extraction	Separation techniques through membranes [50,53]	
Tocopherols and tocotrienols	Palm fatty acid distillate	treatment with alkyl alcohol and sodium methoxide; distillation under reduced pressure; a cooling step; passage of the filtrate through an ion-exchange column with anionic exchange resin; removal of the solvent; molecular distillation [54,55,56]	
Phenolic antioxidants	Aqueous by-products from the extraction of palm oil	Without solvent; based on simple separation principles [57]	
Pepsin	Cod stomach silage	Ultrafiltration together with concentration, and spray-drying [58]	0.5–1 g/kg
Peptone	Cod stomach and viscera silage	Ultrafiltration together with concentration, and spray-drying [58]	100 g/kg
Polyunsaturated fatty acids	Fish wastes	Distillation, low temperature crystallization, enzymatic methods, urea complexation, alkaline hydrolysis, supercritical fluid extraction, microwave assisted extraction	
Collagen	Fish skin, bones and fins	Acid treatment of the by-products	
Gelatin	Fish skin, bones and fins	Heat denaturation of collagen	
Lard	Clean tissues of healthy pigs		
Tallow	Fatty tissues of cattle or sheep		

Supercritical fluid extraction can be conveniently applied to a number of applications. It is suitable for removing 97% of the caffeine from green tea leaves without significantly impacting the valuable catechins and flavonols [42], or essential oils (matricine, chamazulene and α-bisabolol) from chamomile. In this case, the separation between cuticular waxes and essential oil was achieved by applying two separation steps: a 1st separation, performed at 40 bar and 15 °C, thus giving an extract containing only 2.49% of matricine, no chamazulene or α-bisabolol; and a 2nd separation at atmospheric pressure and 0 °C, affording 28.08% of matricine, 0.05% of chamazulene and 2.68% of α-bisabolol [43]. Capsaicinoids and colour components can be extracted from chili pepper in the CO_2_ density range of approximately 625 kg/m^3^ (100 bar, 40 °C). The extraction efficiency of capsaicinoids was 66% and 86% at 40 and 80 °C, respectively. The extraction efficiency of color components was 26% and 34% at 40 and 80 °C, respectively [44].

Rice bran is a matrix rich in colors, flavors, fibers, niacin, thiamin, vitamin B6, iron, phosphorus, magnesium, and potassium. It also includes an oil fraction, known for its hypocholesterolaemic properties, which is commercially extracted with hexane. Perretti *et al.* [45] have successfully applied supercritical fluid to the extraction of oil from rice bran. The extraction conducted at 10,000 psi and 80 °C with only 100 g of CO_2_ gave the highest extraction yield (24.65%). Decreasing temperatures and pressures require a higher amount of CO_2_. γ-Oryzanol can be conveniently extracted from rice bran using ethanol (76%–100%) and temperatures in the range 69–90 °C, giving a yield of 1527–4164 mg/kg [46].

The traditional methods for the extraction of β-glucans from barley brans involve the following steps: inactivation of the endogenous enzymes in the grain; extraction with water or alkali solutions; use of hydrolytic enzymes to remove protein and starch; precipitation of β-glucans and freeze-drying of the solutions [47].

The extraction of lignans from flax seeds can proceed according to a solid-liquid extraction by means of aqueous methanol or ethanol after defatting with hexane. The resulting crude extract undergoes an alkaline treatment with NaOH/KOH 1N [48].

Phenolic acids can be conventionally-extracted from wheat brans using hexane, acetone, or methanol at 60 °C or by emerging techniques such as ultrasound-assisted extraction, using ethanol 64% as the extracting solvent, temperature 60 °C, time 25 min, and microwave-assisted method using methanol as solvent and performing the extraction at 120 °C [23,49].

Besides the oil, by-products of palm oil milling and refining contain nutraceutical molecules. Tocopherols, tocotrienols, sterols, and squalene (2128–13,504 mg/kg) are concentrated in the palm fatty acid distillate deriving from the physical refining of palm oil [50,51,52]. Sterols (4500–8500 mg/kg), tocopherols and tocotrienols (2400–3500 mg/kg), carotenoids (4000–6000 mg/kg), phospholipids, squalene (1000–1800 mg/kg), and phenolics are contained in the palm pressed fibers. Water-soluble palm phenolics can be successfully extracted from aqueous by-products generated from the mill [50,53]. A method was patented for the production of nutraceuticals from palm fatty acid distillate including the treatment with alkyl alcohol and sodium methoxide to convert free fatty acids and glycerides into alkyl esters; the removal of the alkyl esters by distillation under reduced pressure, leaving tocopherols, tocotrienols, sterols, and squalene in the residue; a cooling step to crystallize and separate the higher-melting sterols from tocopherols and tocotrienols; the passage of the filtrate containing tocopherols, tocotrienols and squalene through an ion-exchange column with anionic exchange resin to remove the squalene and produce a concentrated tocopherol and tocotrienol fraction in the solvent; the removal of the solvent; a molecular distillation [54,55,56]. The extraction of palm oil requires the addition of water at certain stages. The result is aqueous by-products containing palm-derived water-soluble extracts with potent antioxidative properties. The Malaysian Palm Oil Board patented a process for extracting phenolic antioxidants, which is solvent free and based on simple separation principles [57]. The process system included a 3-phase decanter system which pellets the suspended solids and floats the oil; a membrane that removes residual oil not removed by the decanter system, an ion-exchange membrane that removes ionic contaminants such as iron, and a molecular weight cut-off membrane that removes the high-molecular-weight components, with the production of a filtrate rich in phenolic antioxidants, *i.e.*, flavonoids, polyphenols and phenolic acids.

## 4. Recovery of Biomolecules from Animal Wastes

Animal-derived processing wastes include: wastes from bred animals such as carcasses, hides, hoofs, heads, feathers, manure, offal, viscera, bones, fat and meat trimmings, blood and other fluids, off-spec animals and meat; wastes from seafood such as off-spec, by-catch, and rubbish from fishing operations, skins, bones, cuttings, viscera, oils, and blood; wastes from dairy processing such as curd, whey, and milk sludge from the separation process [16].

The United States considers everything produced by or from the animal, except dressed meat, to be by-products (edible and inedible). The edible by-products include: blood, meat trimmed from the head, edible fats obtained during slaughter (such as the cowl fat surrounding the rumen or stomach, or the cutting fat which is back fat, pork leaf fat or rumen fat), and slaughter. In commercial slaughter house practice as established in the U.K, the offal is divided into red (head, liver, heart, lungs, tongue, tail *etc.*) and white (fat), plus the set of guts and bladder, the set of tripe (rumen), and the four feet and trimmings. The list originally included the spinal cord and brain, but these are now banned for food use since the outbreak of Bovine Spongiform Encephalopathy (BSE, popularly known as Mad Cow Disease). Some by-products cannot be used in uncooked products. Examples of these items are: blood and blood plasma, feet, large and small intestines, lungs, esophagus meat, rectum, stomach (non-ruminants), first, second and fourth stomach (ruminants), testicles and udder, and poultry parts such as gizzards and necks [59]. Lard is the fat rendered from the clean tissues of healthy pigs while tallow is hard fat rendered from the fatty tissues of cattle or sheep. They can be obtained by dry or wet rendering. In the wet rendering, the fatty tissues are heated in the presence of water, at a low temperature. The quality of the lard or tallow from this process is better than that of products from dry rendering [59]. The information contained in this section is summarized in Table 2.

Today there is an increasing demand for fish raw materials, which promotes better recovery and utilization of the by-products as raw materials for food, nutraceutical, pharmaceutical, and biotechnological applications. Interesting by-products are represented by fish stomachs and viscera silage and fish sauce. Carnivorous fishes have high stomach pepsin contents, and a silage made from minced viscera, or even better, from the separated stomach, will solubilize completely after a few days of storage. By ultrafiltration, concentration, and spray-drying, a cod stomach silage can give a pepsin preparation corresponding to 0.5–1 g pure pepsin per kg, while silages of both stomach and viscera provide about 100 g peptone per kg raw material [58].

Fish oil from fish processing waste, marine fish and chicken visceral wastes are rich sources of polyunsaturated fatty acid concentrates and, in particular, of omega-3 essential fatty acids. Polyunsaturated fatty acids have lower volatility than small-chained fatty acids and hence can be separated effectively by distillation. They have high boiling points, hence molecular distillation is the preferred way of separation. Nevertheless, other technologies can be applied such as low temperature crystallization, enzymatic methods, urea complexation, alkaline hydrolysis, supercritical fluid extraction, microwave assisted extraction, *etc.* Fish skin, but also bones and fins, are potential sources of collagen and gelatin. Collagen is obtained by acid treatment of the by-products whereas gelatin is obtained when collagen undergoes heat denaturation. Gelatin is derived from fish skin by enzymatic hydrolysis.

## 5. Food and Food Processing Uses of the Recovered Biomolecules and By-Products

A wide range of nutraceuticals from various natural sources can be included in extruded products. In particular, four types of compounds deserve to be mentioned: γ-oryzanol from rice bran, which is a potent antioxidant, a cholesterol reducing agent, a tumor inhibiting agent, and a preventing agent in menopausal syndrome treatment [60]; β-glucans extracted from barley flour, which improve lipid metabolism, reduce the glycaemic index, and lower plasma cholesterol [47]; lignan concentrates from flaxseed, which act as anti-cancer, antioxidant, antibacterial, antiviral, and anti-inflammatory agents [61]; and phenolic compound extracted from cereal brans, which antioxidants provide resistance against free radical damage, cancer and cardiovascular diseases [62]. γ-Oryzanol is a heat labile component and its levels decrease with the increase of the extrusion temperature. Ohtsubo *et al.* [63] revealed that extruded and puffed pre-germinated brown rice contained more oryzanol than the unextruded polished rice. Successful incorporation of various fractions of β-glucans into products such as pasta, noodles, breakfast cereals, and dairy products was achieved. Pearled barley, if enriched with β-glucans, can be incorporated into durum wheat semolina to give a pasta that exhibits good cooking quality [45]. Flaxseed or flaxseed meal rich in lignans can be conveniently added to various cereal-based formulations like bread, muffins and other bakery products. According to Viscidi *et al.* [64], in the production of rolled oats, phenolic compounds derived from natural sources such as benzoin, catechin, chlorogenic acid, and ferulic acid can be mixed with the other ingredients prior to extrusion in order to obtain products more resistant to oxidation (retardation of hexanal formation). The final products have a higher phenolic content that the conventional ones, although processing results in a 24%–26% reduction of the amount of the phenolic compounds added.

Pectins can be found in most fruit pomaces and, after extraction and purification, can be added as gelling agents in numerous food products such as jams, fillings, sweets, *etc.* Pomace can also provide other food additives including dietary fibres, lactic acid, pigments, vinegar, natural sweeteners and cellulose [65]. Some tropical fruits contain protein-degrading enzymes (papain in papaya, or bromelain in pineapple) usable as meat tenderisers or in washing powders or beer brewing.

Pectins are classified on the base on their degree of methylation in high-methoxy pectins (having a degree of methylation >50%) and low-methoxy pectins (with a degree of methylation <50%). They are used as gelling agents to impart a gelled texture to fruit-based foods. High-methoxy pectins gel with at least 55% of soluble solids and at pH ≤ 3.5 while low-methoxy pectins gel in the presence of calcium ions [16].

At present, there is a great interest in natural ingredients, which are available from seafood wastes or by-products. The fish pepsin mentioned in the previous section [51] can be used as a rennet substitute in cheese production [66]. Cod pepsin could be used for de-skinning of herring, but it is difficult to obtain an effective de-skinning without damaging the muscle due to variable skin structure and thickness at the different parts of the fish. Cod pepsin can be successfully used for descaling. A pepsin treatment can be used also for facilitate the release of the roe from connective tissues during production of caviar from trout and salmon [67]. The soluble protein fraction from Alaska pollack, shrimp waste, cod heads and sardines demonstrates a certain inhibitory effect on angiotensin I converting enzyme [68], thus indicating that application of such hydrolysates in food may reduce high blood pressure. Fish sauce is made from small pelagic fish species by-products or by-catch fish by salt fermentation. It is obtained as an amber protein solution by tank storage of a fish-salt mixture (3:1) at tropical temperatures for at least a half year [48]. Fish sauce is a source of essential amino acids and can be introduced in the diet of vegetarians to provide a good balance to most vegetable proteins. Furthermore, fish sauce seems to stimulate the proliferation of human white blood cells [69]. Wastes from cultured eel, catfish, milkfish, and ocean-caught tuna, sardine, and squid are sources of fish oil, which can be obtained by supercritical fluid extraction, microencapsulation technology, and enzyme treatment [70].

The modern diet is insufficient in omega fatty acids, hence the intake of oily fish two to three times a week is recommended. Incorporation of fish oils into normal food ingredients, which become can be considered as an alternative in order to increase the intake of polyunsaturated fatty acids, especially eicosapentaenoic and docosahexaenoic acids. Nevertheless, polyunsaturated compounds are highly prone to oxidative deterioration and production of volatile secondary oxidation products and also have undesired fishy smell and off-flavors, which have detrimental effects on consumer acceptance. Microencapsulation can prevent these negative issues since microencapsulated fish oils can be converted to stable dried powdered form and the wall materials of the microcapsules protect the core materials from environmental effects of temperature, humidity, light and reduce its sensorial impact [71]. Interesting amounts of eicosapentaenoic and docosahexaenoic acids can be produced by the use of algae. Perretti *et al.* [72] extracted oil from the algae *Isochrysis galbana* Parke using supercritical carbon dioxide with ethanol as co-solvent. Extraction yield was compared with traditional organic solvents Soxhlet extraction (hexane, petroleum ether, chloroform/ethanol). Extraction yields of 4%–10%, 5%–11%, 15%–28%, 5%–16%, and 5%–15% were obtained by using neat carbon dioxide at 690 bar and 40 °C, carbon dioxide/ethanol, chloroform/ethanol 1/1 (v/v), hexane, and petroleum ether, respectively.

Animal blood has a high level of protein and heme iron and can be considered as an important edible by-product [73] if it has been removed by bleeding an animal that has been inspected and passed for use in meat food products. It has long been used to make blood sausages, blood pudding, biscuits and bread (in Europe), and in blood curd, blood cake and blood pudding in Asia [74]. Although the belief that blood can provide pathogens and toxic metabolites is genuine, consumers must considered that all foods of animal origin are easily contaminated with spoilage microorganisms and possibly pathogens. If blood is taken from a healthy animal, it is essentially sterile. The use of closed-draining systems is suitable to avoid contamination during collection. The transportation in refrigerated isothermal stainless steel trucks to the processing plant prevents re-contamination. Successively, a spray-drying treatment performed at high temperatures inactivates any pathogens eventually present. Tallow and lard can be used for production of margarine and shortening [75], for deep frying [76], and in sausages or emulsified products [77].

## 6. Medicinal and Pharmaceutical Uses of the Recovered Biomolecules and By-Products

A number of medicinal and pharmaceutical uses can be described for recovered molecules and by-products. Some examples are reported in the following paragraphs.

Hot-water extract of pulverized oyster shell produces polypeptides having tyrosinase inhibitory activity (an index for skin-whitening effects) while CaCO_3_ from oyster shells can be used as a calcium supplement. Shrimp and crab shells can be recovered to manufacture chitosan, a “fat-binder” used for weight management to enhance binding of bile acid and excretion of sterols, and thus, lowering cholesterol, and as soluble dietary fiber to improve gastrointestinal function. Astaxanthin, the chromophore of shrimp shells, can be extracted and used as an antioxidant, since it has an efficacy 500 times higher than that of vitamin E, and as regulator of the plasma HDL-cholesterol level [70].

Collagen can have nutraceutical and bio-medical applications since microporous collagen films are used for delivery of anticancer drugs and collagen matrices are used as gene delivery agents that promote bone and cartilage formation. It can be ingested by patients suffering from osteoarthritis and/or osteoporosis. Gelatin is found to be potent antioxidant and antihypertensive and to enhance dietary calcium absorption.

Animal glands and organs such as liver, lungs, pituitary, thyroid, pancreas, stomach, parathyroid, adrenal, kidney, corpus luteum, ovary, and follicle are traditionally used as medicine in many countries. Brains, nervous systems and spinal cords are a source of cholesterol, which is used for the synthesis of vitamin D3. Melatonin, extracted from the pineal gland, is used for the treatment of schizophrenia, insomnia, and other mental diseases. Bile, obtained from the gall bladder, can be used for the treatment of indigestion, constipation, and bile tract disorders. Prednisone and cortisone can be extracted separately from bile and used as medicines. Heparin can be extracted from the liver. Progesterone and estrogen can be extracted from pig ovaries, while relaxin is a hormone extracted from the ovaries of pregnant sows [59]. Purified bovine albumin is used in testing for the Rh factor, in antibiotic sensitivity tests, and as a stabilizer for vaccines [59].

Nutraceuticals extracted from plants and seaweed could be used as appetite modulators. In fact, Mayer *et al.* [78] tested the effects of a nutraceutical product composed of *Garcinia cambogia*, l-carnitine, and a seaweed extract of *Ascophyllum nodosum* on satiety sensations and food preferences in healthy people. They found the absence of differences in energy intake between the group of people who receive the treatment and the other who received a placebo, but they also highlighted a significant reduction in subjective hunger sensations and an increase in satiety and fullness.

## 7. Future Trends

In order to increase the eco-sustainability of food processing industry, it is necessary to exploit co-products before they become wastes. Nevertheless, the reuse of components of co-products is still in its infancy due the necessity to invest in research, new recovery technologies, and/or new production lines, whose costs are still higher than landfill taxes currently in force and composting costs. The only directions in which food industry is focusing its efforts are the reduction of the energy and water consumption, and, in a residual way, the recovery of energy from waste [9].

Exploitation of the co-products can take advantage of greater collaboration between research and industry, adaptation and use of already existing technologies and plants for the recovery of biomolecules from co-products, and the application of economies of scale needed for the treatment of large volumes of biomass characterized by low specific economic values.

In order to increase the reuse of components of co-products, it will be necessary to use novel extraction technologies capable of reducing solvent consumption and hence capable of increasing the overall eco-sustainability of the food life cycle. Moreover, with the aim of increasing the consumer propensity to purchase food products containing recovered biomolecules, it will important to reduce the prices of the enriched foods and also to increase their sensorial acceptance.
